# A Call for the Purpose of Organizing a Tri-State Medical Association

**Published:** 1889-09

**Authors:** Frank Trester Smith

**Affiliations:** Secretary of Committee; Chattanooga, Tenn.


CHATTANOOGA, TENN., August 8, 1889.

DEAR DOCTOR:

The following call has been issued:

"The members of the medical profession in Alabama, Georgia and Tennessee are requested to meet in Chattanooga on the third Tuesday in October, for the purpose of forming a Tri-State Medical Association. All will be admitted to the meeting of the Association, but the membership will be restricted to graduates of regular Medical Colleges in good standing."

This call is signed by committees from Jackson County, Alabama, Medical Society; Chattanooga, Tennessee, Medical Society; Cleveland, Tennessee, Medical Society; Cartersville, Georgia, Medical Society; Dalton, Georgia, Medical Society.

It is hoped that there will be a general turnout of the profession. Papers of interest have been promised by prominent men.

This organization will be independent of all other societies. It will be an association of individual members of the profession of medicine, and will be managed in the interest of medical progress. You are earnestly requested to be present and participate in the exercises.

The session will continue two days. If you desire to read a paper or exhibit a specimen, please notify the undersigned at an early date.

Another circular will be issued in due time announcing the titles and authors of papers.

Fraternally yours,

FRANK TRESTER SMITH, M. D.,
Secretary of Committee.

